# Transcranial slow oscillation stimulation during NREM sleep enhances acquisition of the radial maze task and modulates cortical network activity in rats

**DOI:** 10.3389/fnbeh.2013.00220

**Published:** 2014-01-08

**Authors:** Sonja Binder, Julia Rawohl, Jan Born, Lisa Marshall

**Affiliations:** ^1^Department of Neuroendocrinology, University of LübeckLübeck, Germany; ^2^Institute of Medical Psychology and Behavioral Neurobiology, University of TübingenTübingen, Germany; ^3^Graduate School for Computing in Medicine and Life Sciences, University of LübeckLübeck, Germany

**Keywords:** slow oscillation stimulation, tDCS, EEG, sleep, consolidation, reference memory, working memory

## Abstract

Slow wave sleep, hallmarked by the occurrence of slow oscillations (SO), plays an important role for the consolidation of hippocampus-dependent memories. Transcranial stimulation by weak electric currents oscillating at the endogenous SO frequency (SO-tDCS) during post-learning sleep was previously shown by us to boost SO activity and improve the consolidation of hippocampus-dependent memory in human subjects. Here, we aimed at replicating and extending these results to a rodent model. Rats were trained for 12 days at the beginning of their inactive phase in the reference memory version of the radial arm maze. In a between subjects design, animals received SO-tDCS over prefrontal cortex (PFC) or sham stimulation within a time frame of 1 h during subsequent non-rapid eye movement (NREM) sleep. Applied over multiple daily sessions SO-tDCS impacted cortical network activity as measured by EEG and behavior: at the EEG level, SO-tDCS enhanced post-stimulation upper delta (2–4 Hz) activity whereby the first stimulations of each day were preferentially affected. Furthermore, commencing on day 8, SO-tDCS acutely decreased theta activity indicating long-term effects on cortical networks. Behaviorally, working memory for baited maze arms was enhanced up to day 4, indicating enhanced consolidation of task-inherent rules, while reference memory errors did not differ between groups. Taken together, we could show here for the first time an effect of SO-tDCS during NREM sleep on cognitive functions and on cortical activity in a rodent model.

## Introduction

Sleep plays an important role for the consolidation in several memory systems in humans as well as in rodents (Plihal and Born, 1997; Stickgold, 2005; Diekelmann and Born, 2010). According to the trace transformation theory (Winocur et al., 2010), an active systems consolidation takes place: for hippocampus-dependent memories, it is assumed that core features (the “gist”) of a memory is temporally stored in the hippocampus and become gradually independent of the hippocampus over the course of consolidation, largely taking place during sleep (Frankland and Bontempi, 2005; Rasch et al., 2007; Diekelmann and Born, 2010; Inostroza and Born, 2013). Within this framework, reactivation of neural ensembles during hippocampal sharp-wave ripples are the electrophysiological events associated with hippocampo-neocortical communication and redistribution of memory representations for long-term maintenance (Buzsaki, 1996; Peyrache et al., 2009; Wierzynski et al., 2009; Lesburgueres et al., 2011). Selective suppression of hippocampal ripples after daily training in the radial arm maze impaired reference memory (Girardeau et al., 2009). The cortical sleep slow oscillation (SO) occurring during slow wave sleep is thought to provide a temporal time frame for the hippocampal-neocortical dialog to take place (Sirota et al., 2003; Mölle et al., 2006). The SO with its UP- and DOWN states is coupled in time to hippocampal sharp-wave ripples as well as to sleep spindles, two brain rhythms also closely associated with sleep-dependent memory consolidation (Mölle et al., 2009; Andrillon et al., 2011; Fogel and Smith, 2011; Girardeau and Zugaro, 2011).

It is now well established that weak electric fields, either externally applied or generated endogenously by oscillating neural networks, are capable of modulating neuronal activity (e.g., Bindman et al., 1962; Francis et al., 2003; Deans et al., 2007; Anastassiou et al., 2010; Weiss and Faber, 2010; Buzsaki et al., 2012). Studies on sleep-related rhythms have shown that such oscillating weak electric fields can entrain neuronal activity to the applied frequency; with these effects relying critically on brain state, i.e., being most effective if matching in frequency to the prevailing endogenous rhythms (Fröhlich and McCormick, 2010; Ozen et al., 2010; Marshall and Born, 2011; Marshall et al., 2011; Ali et al., 2013). Transcranial DC stimulation at the frequency of endogenous SO (SO-tDCS) during non-rapid-eye movement (NREM) sleep in humans not only boosted endogenous SO and spindle activity, but additionally improved memory consolidation and/or learning capacity (Marshall et al., 2006; Antonenko et al., 2013) for a hippocampus dependent task, underscoring the importance of SO for these processes.

The impact of weak oscillatory electric stimulation during sleep on memory consolidation has to our knowledge not been investigated yet in a rodent model. The development of such models is however necessary to further elucidate the underlying mechanisms of endogenous and external weak electric fields, their impact on neural networks and their relevance for learning and memory. To address these questions, we investigated the influence of SO-tDCS applied on multiple days during post-training NREM sleep on EEG activity and memory performance in rats. We used the reference memory version of the radial maze task (Jarrard, 1983), to examine putative effects of SO-tDCS on EEG activity on a short-term (i.e., immediately following stimulation) as well as on a long-term (i.e., over consecutive days of stimulation) time scale, and to assess its impact in parallel on both reference memory and working memory. We hypothesized SO-tDCS would enhance task performance and endogenous activity within the SO frequency range, the latter most pronounced immediately following the initial stimulation(s) of each day, thereby essentially replicating the results of Marshall et al. (2006) in a rat model.

## Materials and methods

### Animals

Twenty two male Long Evans rats (Janvier, Le Genest-Saint-Isle, France), 10–11 weeks old and weighting 306 ± 3 g at time of surgery, were used. Before surgery, animals were housed individually in Standard type IV Macrolon cages with *ad libitum* access to food and water under a 12 h/12 h light-dark cycle (lights-on 07.00 AM) and were handled daily for 5 min on 7 consecutive days. All experimental procedures were performed in accordance with the European animal protection laws and policies (directive 86/609, 1986, European Community) and were approved by the Schleswig-Holstein state authority.

### Surgery

Animals were anesthetized with isoflurane (induction: 3.5 ml/min in 700 ml/min O_2_, maintenance: 1.3–1.9 ml/min in 700 ml/min O_2_). Additionally, 0.6 mg/kg medetomidine (Dorbene, Dr. E. Graeub AG, Switzerland) was given i.p. for intrasurgical pain relief and 0.04 mg/kg atropin (Atropinum Sulfuricum, Eifelfango, Germany) s.c. to prevent breathing problems. For epidural EEG recording a stainless steel screw-electrode (diameter 1.57 mm, shaft length 2.4 mm, Plastics One, USA) was placed over the left frontal cortex (AP: + 1.7 mm, L: −0.5 mm) and referenced to an occipital site (AP: −12.0 mm; L ± 0.0 mm). For bilateral stimulation screw-electrodes of the same size as above were drilled halfway through the skull. Anodes for SO-tDCS were positioned bilaterally over the prefrontal cortex (PFC) (AP: +3.9 mm, L: ±2.0 mm) and the return electrodes over the cerebellum (AP: −10.0 mm, L: ±2.0 mm). Two holding screws were positioned over the right somatosensory cortex (AP −4.0 mm, L: +2.0 mm), another anterior electrode (AP: +6.9, L: +1.1) was used as ground. For EMG recordings, two insulated stainless steel wire electrodes (Plastics One, USA) were implanted bilaterally in the neck muscles. All electrodes were connected to two plastic pedestals (Plastics One, USA), one for polysomnographic recording and one for SO-tDCS, covered with adhesive luting dental cement to enable long-term stability on the skull (C and B MetaBond, Parkell Inc, USA) and finally fixed with cold polymerizing dental resin (Palapress, Heraeus Kulzer GmbH, Germany). Following surgery, rats were given 1 mg/kg atipamezol (Alzane, Dr. E. Graeub AG, Switzerland) i.p. to antagonize the effects of medetomidine, 5 mg/kg caprofen (Rimadyl, Pfizer AG, Switzerland) i.p. for pain relief and 5 ml 0.9% NaCl-solution s.c. for fluid substitution. Animals had 7 days for recovery from surgery before moving to the experimental room.

### Radial maze apparatus and experimental setting

The experimental room was divided by a curtain and light wood walls into three areas: one housing/recording area, one radial maze area and one observation area for the experimenter. The radial maze was made of black PVC with eight arms (L 40 cm, W 9 cm) radiating from a central platform (diameter 24 cm). The arms were enclosed with 17-cm high walls, one of them made of black PVC, the other wall and the end wall made of transparent Plexiglas. At a recess at the end of each arm a glass cup which served as a food well, was inserted. The central platform was separated from the arms by 30 cm high doors, which could be mechanically operated from the observation area. The whole maze was positioned 50 cm above the ground and a camera was mounted above the apparatus. Surrounding furniture and posters affixed to the walls served the animal as extra maze cues for spatial orientation. Below the end of each arm a cup containing bait food was placed to impede orientation on potential olfactory cues.

### Experimental procedure and design

Following surgery, animals were housed in recording boxes (35 × 35 × 46 cm), made of dark-gray PVC and containing Plexiglas-windows on two opposite sides for visual contact to the neighboring box. Food restriction started when animals reached their pre-surgical weight or latest on the 6th post-surgical day. During the course of the whole experiment, rats were kept between 85 and 90% of presurgical bodyweight and were weighed daily. On the 6th post-surgical day, they were moved to the housing area in the experimental room where they stayed until the end of the experiment. All procedures described beneath were conducted between 8 AM and 1 PM if not stated otherwise. Animals were randomly assigned to the SO-tDCS (STIM, *N* = 11) or the control group (SHAM, *N* = 11). On the 7th post-surgical day, a 2-h habituation recording was conducted to adapt the animals to the recording conditions. On the 8th post-surgical day, a 2-h baseline recording was conducted, and on day 9 a baseline stimulation recording took place to check for signal quality and proper functioning of the whole setup. One day later, animals were habituated to the radial maze. For this purpose, 16 food baits (Choco Krispies, Kellogg GmbH, Germany) were scattered throughout the apparatus. The animal was placed on the central platform (doors closed), after ~5 s the doors were opened and the animal had the possibility to explore the maze until all baits were eaten or 20 min had elapsed. If after this time the animal had not consumed any of the bait, time was prolonged by 5 min. Afterwards the animal was connected to the recording and SO-tDCS cables, placed back in its box and recorded for 2 h. Here, no SO-tDCS was applied. On the following day, the experiment proper started. During 12 consecutive days, each animal received 3 trials in the radial maze per day, separated by 3 min the animal spent on the central platform with doors to the arms closed. Every day, the same three arms (with reference to positioning of the maze in the room and external cues) were baited. To prevent the use of intra-maze cues, the maze was rotated daily by 45°. A trial ended when the animal found and consumed all baits or after 3 min. After 3 trials, animals were connected to the cables and recorded for 2 h. Between animals, the maze was thoroughly cleaned with 60% ethanol solution.

### Sleep recordings

Six recording boxes were placed in the experimental room. The polysomnographic electrodes were connected through a swiveling commutator (Plastics One, USA), allowing free movement inside the box, to a Grass Model 15A54 amplifier (Grass Technologies, AstroMed GmbH, Germany) in an adjacent room. EEG and EMG signals were amplified, filtered (EEG: high pass 0.01 Hz, low pass 300 Hz; EMG: high pass 30 Hz, low pass 300 Hz, −6 dB cutoff frequency and at least −12 dB per octave roll-off), subsequently digitized at a sampling rate of 1000 Hz (CED 1401, Cambridge Electronics, UK), recorded using Spike2 software (Cambridge Electronics, UK) and stored on hard disk. The animals could be visually monitored on a PC monitor in the adjacent room via cameras mounted above the recording boxes.

### Stimulation parameters

The SO-tDCS electrodes (see section Surgery) were connected through the same swiveling commutator as the EEG and EMG, but through a separate cable to a battery driven constant current stimulator in the adjacent room. A sinusoidal constant current fluctuating between 0 and 5.6 μA at a frequency in the range of slow oscillation (1.5 Hz) was applied. Current was bilaterally synchronized.

Stimulation started after the first occurrence of 60 s stable NREM sleep and always lasted for 30 s, followed by a stimulation free interval of at least 30 s. If the animals showed signs of awakening during stimulation (movement and/or increased EMG activity), or if the animal showed any sleep stage change during the stimulation free period, again 60 s of stable NREM sleep was awaited before the next stimulation started. Animals received stimulations for 1 h, starting with the time of the first stimulation. In the SHAM condition, no stimulation was applied, but the EEG record was marked at the respective intervals.

### Data reduction and statistical analyses

#### Behavioral measures

Arm entries, consumption of baits and trial duration were scored online by the experimenter. An arm entry was scored if the animal entered an arm with all four paws. Following measures were computed: (i) reference memory errors (entries into arms which never contained a bait), and (ii) working memory errors (re-entries into arms already visited during the ongoing trial). The latter were further divided into (iii) working memory errors for baited arms and (iv) working memory errors for never baited arms. The mean values over the 3 consecutive trials/day of all measures were subjected to statistical analysis.

#### Sleep architecture

Sleep architecture was determined from the EEG and EMG during the 2 h of daily recording using 10-s epochs for scoring according to standard criteria (Neckelmann et al., 1994) with the software SleepSign for Animal (Kissei Comtec, Japan).

In short, “waking” (W) was identified by sustained EMG activity and mixed-frequency EEG, “NREM sleep” by low EMG, high-amplitude low-frequency EEG with a high proportion of delta activity, “Pre-rapid eye movement sleep” (PreREM sleep) by low EMG and high-amplitude EEG spindle activity, and “REM sleep” by a further reduced EMG-signal and low-amplitude EEG with high theta (5–9 Hz) activity. Stimulation epochs were scored as a separate “stage” (STIM or SHAM), because a reliable assignment to a distinctive sleep stage was not always possible due to massive signal distortion. For the SHAM group without stimulation, sham-stimulation intervals were inserted according to the same rules as for real stimulation, i.e., sham-stimulation started after 60 s of stable NREM sleep and each sham stimulation lasted 30 s with a 30 s sham-stimulation free interval. If the animal showed any sleep stage change during the stimulation free period, again 60 s of stable NREM sleep were awaited before the next sham-stimulation started. Regarding sleep architecture, following measures were computed: total sleep time (TST), duration of the different stages (W, NREM, REM, PreREM sleep) in minutes and as percentage of TST. Furthermore, sleep latency (start of recording to first occurrence of stable NREM sleep), REM sleep latency and the number of stimulations were computed.

#### EEG analysis

Before power spectral analyses, EEG data were first low pass filtered (FIR filter, 35 Hz). Subsequently, a Hanning window was applied on blocks of 8192 sample points (~8.2 s) of EEG data before power spectra were calculated using Fast Fourier Transformations (FFT). Generally, data was normalized indicating the percentage of each bin (bin size 0.12 Hz) with reference to the total spectral power between 0.85 and 35 Hz. To account for possible violations of the assumption of normal distribution, the normalized data was log transformed as proposed by Gasser et al. (1982): (log(*x*/[1−*x*]), where log refers to the natural logarithm and x represents the relative power in a given frequency band. For statistical analyses, these transformed values were used. Analyses were conducted for (i) all NREM sleep and (ii) all REM sleep epochs of the baseline and the experimental recordings, (iii) during acute (sham)stimulation, and (iv) for the mean of all 10-s stimulation free epochs immediately following stimulation if they consisted of NREM sleep only. The latter analysis was further refined by additionally analyzing just the first and the last 10-s stimulation free interval of the day. This was done due to effects found in a comparable study in human subjects (Marshall et al., 2006), where changes in EEG activity after SO-tDCS were most pronounced in the first stimulation-free intervals. Mean spectral power was calculated for the slow oscillation (SO) band (0.85–2.08 Hz), the upper delta band (2.08–4.03 Hz), theta band (5.00–9.03 Hz) and the spindle band (10.50–13.59 Hz). We split the delta band into SO and upper delta to enable a more detailed analysis as in Binder et al. (2012) and in Achermann and Borbely (1997). Sleep spindles were detected based on the algorithm used by Eschenko et al. (2006). Spindle density was calculated across 1 min intervals of NREM sleep for the same time ranges as for the FFT analyses.

#### Statistics

For behavioral measures, sleep architecture and EEG power analysis ANOVAs for repeated measures were used, followed by *post-hoc* Student's *t*-tests where appropriate. A *P* < 0.05 was considered significant. Results are given as means ± s.e.m. unless indicated otherwise.

## Results

### Behavioral measures

**Figure 1:**
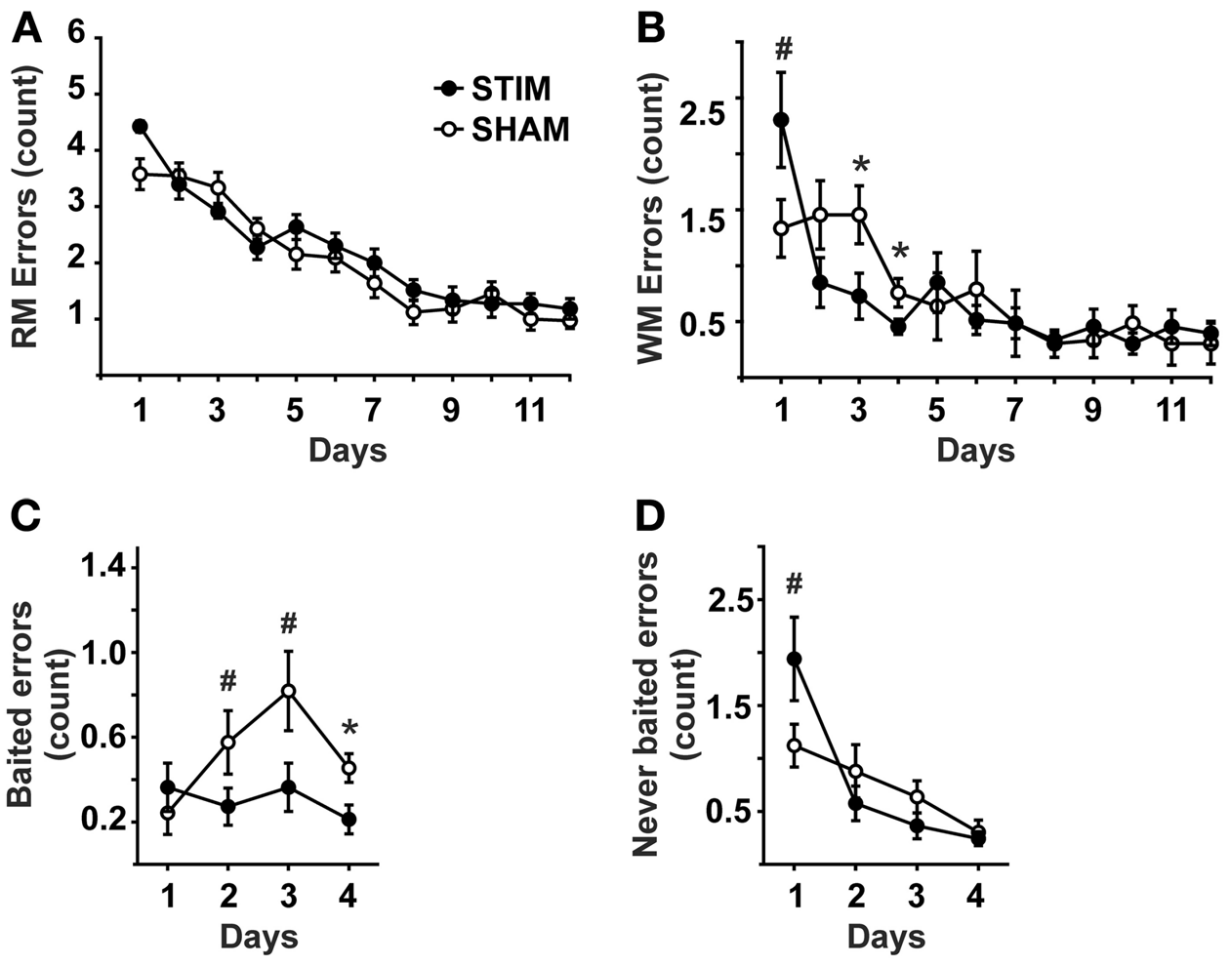
**Behavioral measures (mean ± s.e.m.). (A)** Reference memory errors. **(B)** Working memory errors. **(C)** Working memory errors made on baited arms (= re-entries into baited arms where the bait has been consumed already within the ongoing trial). **(D)** Working memory errors made on never baited arms (= re-entries into never baited arms within the ongoing trial).• Represent STIM condition, ◦ represent SHAM condition. ANOVAs for repeated measures followed by *post-hoc t*-tests. ^*^p < 0.05, ^#^*p* < 0.1.

Measures of behavioral performance are depicted in Figure 1. The number of reference memory errors declined in the course of training from day 1 to 12 [day: *F*_(11, 220)_ = 47.88, *p* < 0.001], without an overall difference between groups nor a significant interaction effect [condition: *F*_(1, 20)_ = 1.06, *p* = 0.316; condition × day: *F*_(11, 220)_ = 1.68, *p* = 0.08].

The number of working memory errors also declined with training [day: *F*_(11, 220)_ = 10.35, *p* < 0.001], without an overall group difference [condition: *F*_(1, 20)_ = 0.82, *p* = 0.778]. However, a significant interaction across days effect was detected [condition × day: *F*_(11, 220)_ = 2.45, *p* = 0.034]. *Post-hoc* tests revealed this effect to be due to a tendency of the STIM group toward poorer performance on day 1, but significantly better performance on subsequent days 3 and 4, (Figure 1B). Since significant differences in working memory errors were found only until day 4, the differential analysis of re-entries into baited and never baited arms was restricted to the first 4 days. Analyses revealed that STIM animals made less errors on baited arms than the animals in the SHAM group [condition: *F*_(1, 20)_ = 7.74, *p* =.011], Figure 1C. For re-entries into never baited arms a differential effect was observed between conditions [condition × day: *F*_(3, 60)_ = 3.52, *p* = 0.033], although both groups revealed an overall decline in errors [condition: *F*_(1, 20)_ = 0.79, *p* = 0.782; day: *F*_(3, 60)_ = 15.24, *p* < 0.001]. *Post-hoc* tests revealed STIM animals tended to perform more poorly on day 1 (Figure 1D).

Habituation trials conducted 1 day prior to the experiment proper did not differ between the groups, neither in regard to the number of consumed baits [STIM: 13.8 ± 1.0, SHAM: 12.2 ± 1.4; *T*_(20)_ = 0.94, *p* = 0.358] nor duration of the trial [STIM: 19.1 ± 1.6 min, SHAM: 18.5 ± 0.6 min; *T*_(20)_ = 0.31, *p* = 760]. Body weight did not differ between the groups [STIM: 86.7 ± 1.7%, SHAM: 87.4 ± 0.7 %; condition: *F*_(1, 20)_ = 0.59,*p* = 0.456; condition × day: *F*_(3.6, 200)_ = 1.81, *p* = 0.143].

### Sleep architecture and stimulation

**Figure 2:**
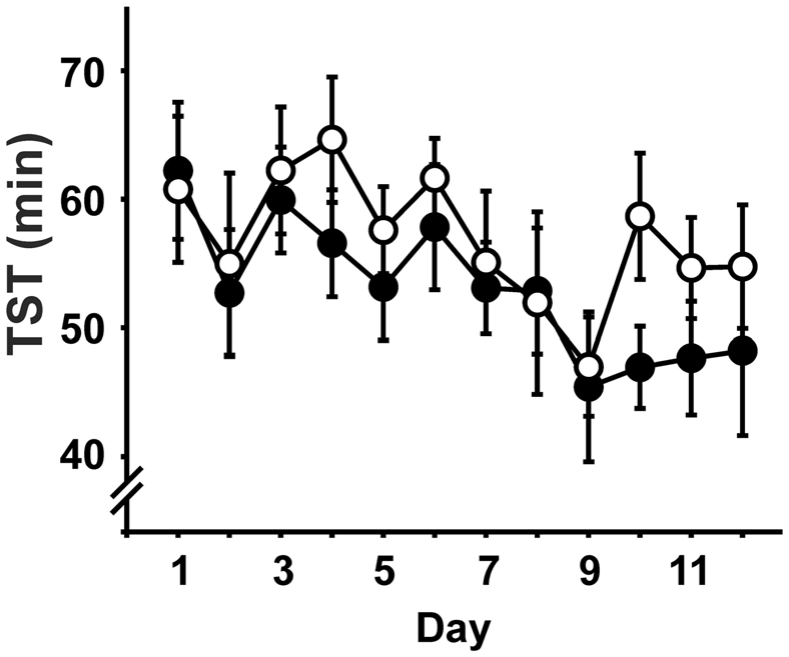
**Total sleep time (TST; mean ± s.e.m.) across the daily 2h-recording period in minutes.** • Represent STIM condition, ◦ represent SHAM condition. There were no differences between the conditions, but TST decreased over experimental days.

In brief, there were no significant differences between the groups regarding any of the measures of sleep architecture and no interactions with day (Time spent awake, in NREM, REM or PreREM sleep, duration of Stim/Sham epochs, all expressed both in minutes and as percentage of TST). During the course of the experiment, the amount of time animals spent awake increased at the expense of all sleep stages [TST: day: *F*_(6.9, 220)_ = 2.98, *p* = 0.006; Figure 2], but sleep latency and REM latency did not change across days (sleep latency: STIM: 19.8 ± 1.1 min, SHAM: 19.7 ± 1.0 min; REM latency: STIM 54.9 ± 1.2 min, SHAM: 53.9 ± 1.5 min; means are given across the experimental days, *p* > 0.05).

SO-tDCS was only applied when animals were in NREM sleep. When signs of awakening as defined in the Methods were evident during the 30 s (sham)stimulation period or sleep stage changed within 10 s after stimulation ended, the EEG-interval was marked and rejected from further analyses. The number of (sham)stimulations did not differ between the conditions, neither if all applied (sham)stimulations were analyzed [STIM: 20.0 ± 1.2, SHAM: 18.3 ± 1.2; condition: *F*_(1, 20)_ = 1.07, *p* = 0.313; condition × day: *F*_(11, 220)_ = 1.52, *p* = 0.126] nor if only percentage of (sham)stimulations without rejected intervals were considered [STIM: 48.0 ± 3.7, SHAM: 50.2 ± 3.7; condition: *F*_(1, 20)_ = 0.18, *p* = 0.677; condition × day: *F*_(11, 220)_ = 0.77, *p* = 0.673].

### EEG analysis

The composition of NREM sleep during the complete 2-h recording period did not differ between the groups in any of the examined frequency bands, neither during baseline recording (Table 1) nor during the subsequent experimental recording sessions (Table 2).

**Table 1:** **Power in relevant frequency bands during baseline recording**.

	**Mean ± s.e.m.**		***T***	***p***
	**STIM**	**SHAM**		
SO (0.85–2 Hz)	27.75 ± 1.51	25.78 ± 1.63	0.91	0.375
Upper delta (2–4 Hz)	26.31 ± 0.77	24.25 ± 2.03	1.07	0.308
Theta (5–9 Hz)	18.95 ± 0.82	19.29 ± 1.06	−0.20	0.846
Spindle (10.5–13.6 Hz)	7.19 ± 0.55	6.84 ± 0.50	0.41	0.686

Means are given in percentage of total power between 0.85 and 35 Hz for descriptive purposes. T-Tests are conducted on logarithmized data. Degrees of freedom: T(20). In case of inhomogeneous variances, values were corrected.

**Table 2:** **F-statistics for EEG power during NREM sleep**.

	**Complete 2 h-recording**		**10 s post-stimulation**	
	***F***	***p***	***F***	***p***
**SO (0.85–2.08 Hz)**				
Condition	2.82	0.109	1.72	0.204
Day	2.73	0.014^*^	1.93	0.071^#^
Condition × day	0.71	0.642	0.70	0.672
**UPPER DELTA (2.08–4.03 Hz)**				
Condition	1.34	0.261	4.18	0.054^#^
Day	1.64	0.089^#^	1.34	0.202
Condition × day	1.56	0.121	1.73	0.074^#^
**THETA (5.00–9.03 Hz)**				
Condition	2.15	0.158	0.82	0.375
Day	2.53	0.005^*^	2.04	0.039^*^
Condition × day	0.75	0.686	0.85	0.595
**SPINDLE (10.50–13.59 Hz)**				
Condition	0.154	0.699	0.89	0.356
Day	2.14	0.033^*^	0.68	0.741
Condition × day	0.01	0.697	1.21	0.283

Degrees of freedom: “condition” *F_(1, 20)_*, “day” and “condition × day” *F_(11, 220)_*; Huynh-Feldt corrections were used if necessary.

* p < 0.05,

# p < 0.1, ANOVAs for repeated measures.

**Figure 3:**
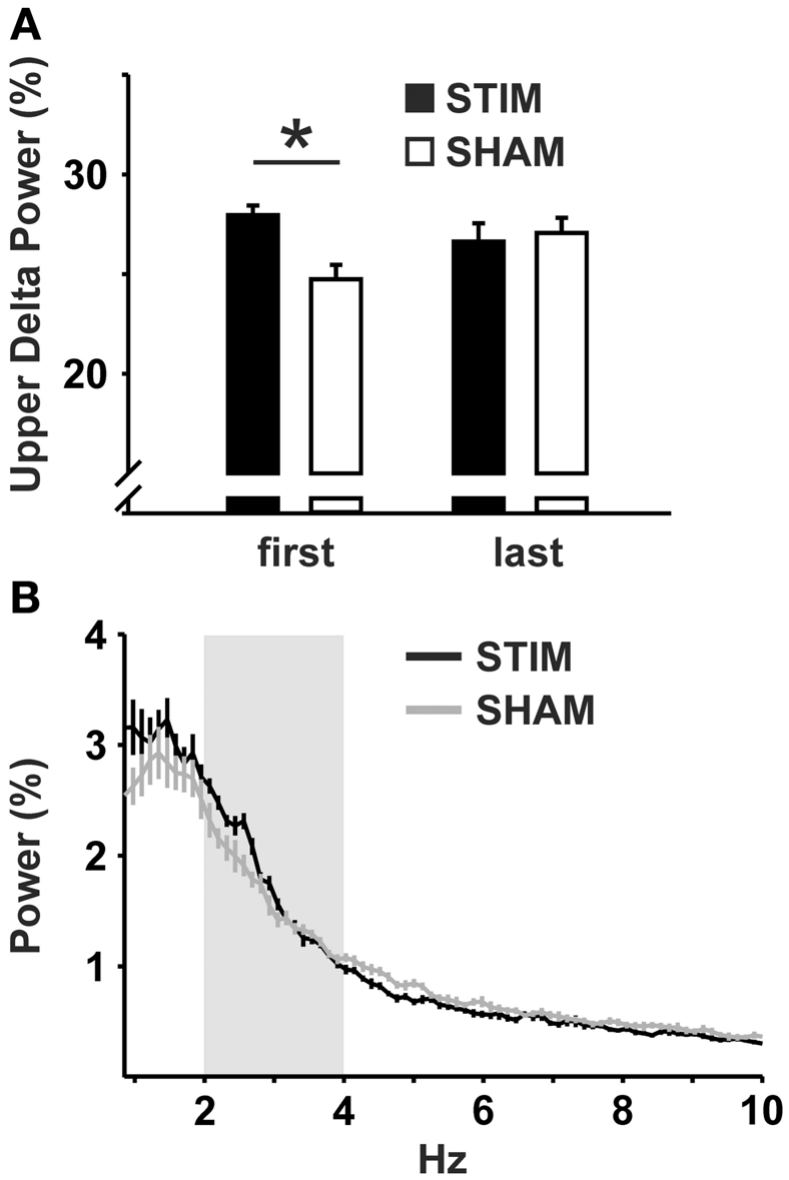
**Upper delta power within the 10 s intervals of post-stimulation NREM sleep. (A)** Mean upper delta power (2.08–4.03 Hz) within the first and the last post-stimulation interval of the day across all 12 experimental days. ANOVAs for repeated measures followed by *post-hoc t*-tests. ^*^p < 0.05. **(B)** Mean power spectra within all 10 s intervals of post-stimulation NREM sleep across all 12 experimental days. Upper delta band is marked in light gray. Note the peak at ~2.5 Hz in the STIM, but not in the SHAM condition.

For the 10 s intervals of post-stimulation NREM sleep a strong trend toward an increased upper delta power in the STIM group was evident (*p* = 0.054; Table 2). To further elaborate on the temporal dynamics of the STIM induced increase in delta power we specifically compared effects of SO-tDCS within the first and last post-stimulation intervals of all 12 days. Figure 3 reveals for upper delta power a significant interaction of “position” (i.e., first vs. last post-stimulation interval of the day) × group (*p* = 0.015). Upper delta power was higher in the STIM group for the first but not the last post-stimulation interval as assessed by ANOVAs conducted separately on the first and last post-stimulation interval [*F*_(1, 20)_ = 5.38, *p* = 0.031 for the effect of group, first interval; Figure 3]. In addition, independent of condition, the first interval compared to the last, revealed lower SO together with higher theta and spindle power, most probably reflecting the deepening of sleep across the 2 h recording [*F*_(1, 20)_ > 9.83, *p* < 0.005 for the main effect of position in the three frequency bands].

**Figure 4:**
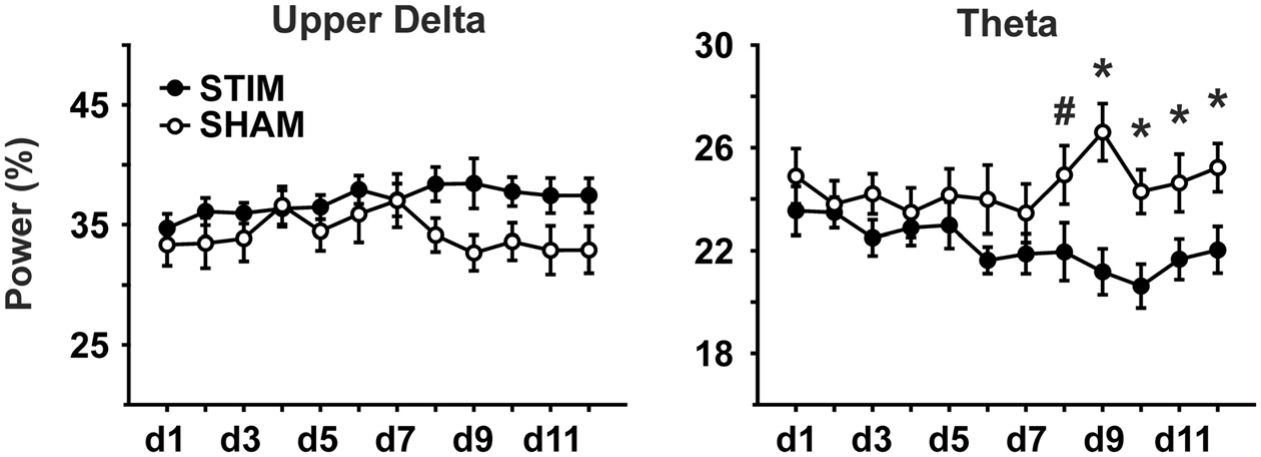
**EEG power during the acute (sham)stimulation.** A significant group difference and an interaction could only be seen for the theta band (5.00–9.03 Hz). Note, SO power could not be analyzed due to frequency overlap with SO-tDCS. ANOVAs for repeated measures followed by *post-hoc t*-tests. ^*^p < 0.05, ^#^*p* < 0.01.

During the 30 s period of acute stimulation (and SHAM stimulation, respectively), FFT analysis revealed from day 8 on significantly lower theta power in the STIM than SHAM group during the stimulation period (effect of condition and interaction *p* < 0.05; Figure 4 and Table 3). A subsequent comparison of these responses during acute stimulation to corresponding values of STIM and SHAM within the baseline recording demonstrated a significant decrease of theta power during days 9 and 10 (*p* < 0.05) and a trend on days 11 and 12 (*p* < 0.1) for the STIM group, with no significant deviations from baseline for the SHAM group (*p* > 0.1).

**Table 3:** **F-statistics for EEG power during acute (sham)stimulation**.

	***F***	***p***
**UPPER DELTA (2.08–4.03 Hz)**		
Condition	2.72	0.114
Day	1.27	0.242
Condition × day	1.40	0.172
**THETA (5.00–9.03 Hz)**		
Condition	4.61	0.044^*^
Day	1.48	0.143
Condition × day	2.92	0.002^*^
**SPINDLE (10.50–13.59 Hz)**		
Condition	0.60	0.449
Day	1.7	0.089^#^
Condition × day	0.92	0.511

Degrees of freedom: “condition” *F_(1, 20)_*, “day” and “condition × day” *F_(11, 220)_*; Huynh-Feldt corrections were used if necessary.

* p < 0.05,

# p < 0.1, ANOVAs for repeated measures.

Sleep spindle density during NREM sleep did not differ between the groups, neither for the whole recording period, nor if only the 10 s post-stimulation intervals or acute stimulation periods were considered (all *p* > 0.05). For REM sleep during the total 2 h-recording period no differences were found for theta power, neither between conditions nor days (all *p* > 0.05).

## Discussion

The present study set out to modulate EEG activity and memory consolidation by applying SO-tDCS during post-training NREM sleep. We had hypothesized that SO-tDCS within NREM sleep would enhance endogenous slow oscillatory brain electric activity and thereby facilitate hippocampus-dependent memory consolidation. Our findings revealed that SO-tDCS did indeed modulate both behavior and endogenous EEG activity in the rat and thereby in essence replicate and extend prior findings in human subjects (Marshall et al., 2006).

Our four main findings are: firstly, post-stimulation EEG responses were most evident for upper delta activity: stimulation increased upper delta activity in the mean over all experimental days. Additionally, a temporal component within the recording session appears to be involved, as the effects of SO-tDCS on upper delta activity were most pronounced at the beginning of the 2 h recording session, i.e., following the first stimulation of the day. Secondly, during the last 4–5 experimental days SO-tDCS significantly reduced the amount of theta activity in comparison to SHAM animals as well as in comparison to the baseline level of the STIM group. Thirdly, at the behavioral level, a component of working memory was also affected by stimulation within the first 4 days, with the stimulation group revealing significantly less re-entries into baited arms. Fourthly, reference memory errors significantly decreased across the 12-day experimental period. However, groups did not differ significantly.

Comparable to prior SO-tDCS studies in human subjects, EEG activity at the beginning of the post-training stimulation intervals was enhanced within the delta range (Marshall et al., 2006; Antonenko et al., 2013). This indicates SO-tDCS was able to influence endogenous EEG activity through resonance effects of the applied oscillatory field on cortical neuronal networks (Francis et al., 2003; Ali et al., 2013). The enhancement in upper delta activity was strongest within the first post-stimulation interval of the day when compared to the last interval, similar to a previous observation (Marshall et al., 2006). Interestingly, in the present experiment in rats upper delta, but not the SO band was enhanced. The arbitrarily division of the delta rage into a SO and upper delta band was done, however, for comparative purposes, and not based on a verified functional division between these two EEG bands. In fact, studies indicate that the phenomenological EEG delta waves probably represent an equivalent of the SO hyperpolarizing phase (Csercsa et al., 2010; Buzsaki et al., 2012). Furthermore, to what extent the increase in upper delta activity can be described by mechanisms responsible for a frequency shift in resonant activity, not uncommon in biological systems (Lau and Zochowski, 2011), needs further investigation.

The decrease in EEG theta during acute stimulation commencing on day 8 may at first seem at odds to the hypothesized facilitatory effect of SO-tDCS on SO and its associated facilitation of memory consolidation during NREM sleep. The occurrence of cortical theta networks during wakefulness is associated with processes of attention, exploration, working memory as well as with encoding and retrieval (Klimesch, 1999; Kawamata et al., 2007; Young and McNaughton, 2009; Nyhus and Curran, 2010; Colgin, 2013). Particularly in rodents, theta rhythm is characteristic of exploratory behavior and REM sleep, but not of NREM sleep where slower frequencies prevail. However, deep NREM sleep is associated with increased power in slow frequencies (as SO) and reduced faster frequencies (as theta; Grasing and Szeto, 1992; Bjorvatn et al., 1998), thus the simplest explanation would be that the observed reduction in theta of the STIM group was related to an increase in induced slower frequencies. In fact, facilitation or entrainment of SO had been expected here, as shown to be induced previously (Marshall et al., 2006; Fröhlich and McCormick, 2010; Ozen et al., 2010). Interestingly, associations between delta and theta rhythm were shown before (Lakatos et al., 2005; Carracedo et al., 2013), as well as relations between presumed *frontal* cortical theta in humans and SO-tDCS (Kirov et al., 2009; Marshall et al., 2011). A direct measurement of SO activity during acute SO-tDCS was however not possible here due to frequency overlap of endogenous SO and SO-tDCS. Also an acute increase in upper delta activity, detectable in mean values, did not reach significance. The delayed occurrence of theta reduction in response to SO-tDCS starting from day 8, in comparison to controls as well as to own baseline, could be interpreted as a kind of plasticity or learning within the cortical network (Shahaf and Marom, 2001; le Feber et al., 2010), even if it was devoid of any presently measured behavioral correlate. The fact that theta activity during acute (sham)stimulation did not differ between groups up to day 8 excludes stimulation artifacts to be responsible for this effect.

On the behavioral level, on days 2–4 working memory errors (i.e., for baited arms) appear decreased in the animal group receiving SO-tDCS during NREM sleep. An interesting finding in the present study is that working memory improvement was limited to the baited arms, i.e., it seems the STIM animals were better in acquiring the rule “if a bait is eaten already it will not be replaced within the same trial” and behave according to this rule. At the morphological level this would indicate that mPFC activity or hippocampal-mPFC interactions were affected: several studies in humans and rodents describe the PFC as the brain region where initially hippocampus-dependent memories, including learned task-inherent rules, are stored for the long term thus implicating this region in memory consolidation (Frankland and Bontempi, 2005; Gais et al., 2007; Leon et al., 2010; Darsaud et al., 2011). It was shown that neural patterns of mPFC activity seen during response selection in a rule learning task are preferentially replayed during subsequent sleep and depended strongly on successful acquisition of the task (Peyrache et al., 2009). As replay events are temporarily highly coupled to the occurrence of the UP state of cortical slow oscillatory activity (Ji and Wilson, 2007; Peyrache et al., 2009), one could hypothesize that enhancement of slow cortical activity by SO-tDCS could have facilitated the hippocampal-mPFC interaction and therefore improved consolidation of task-inherent rules.

On the other hand, the PFC plays also a prominent role as mediator of executive functions by supporting processes associated with working memory, temporal processing of information, acquisition of task rules and decision making (Laroche et al., 2000; Hayton et al., 2010; Kesner and Churchwell, 2011; Velazquez-Zamora et al., 2011). However, if the SO-tDCS during post-learning sleep would have in general positively affected executive functions, an improvement in both components of working memory, i.e., reduced errors for baited and non-baited arms, should have been seen in the STIM animals. In fact a in a study by Joel et al. (1997), mPFC lesions lead to a transient increase of working memory errors for baited arms during early stages of training, and it was suggested that this effect resulted from difficulties to learn a memory-based strategy to solve the task. Thus, superior performance of the STIM group within the first 2–4 days could primarily be attributed to an effect of SO-tDCS on the memory consolidation of task rules involving hippocampo-neocortical network interactions.

The site at which SO-tDCS initially exerted its effect and the underlying neurophysiological mechanisms are however still open. After effects of constant-tDCS, which may also be mediator of plastic effects induced by oscillatory stimulation, have been suggested to involve BDNF, adenosine, calcium influx, NMDA receptor activity, regulation of gene expression and protein synthesis (Gartside, 1968; Islam et al., 1995; Liebetanz et al., 2002; Fritsch et al., 2010; Groppa et al., 2010; Marquez-Ruiz et al., 2012; Ranieri et al., 2012; Ali et al., 2013; Marshall and Binder, 2013; Reato et al., 2013). Pairing of tDCS of the PFC in the rat with training on working memory and skill learning benefited skill retention and spatial working memory (Dockery et al., 2011; de Souza Custodio et al., 2013). More efficient neurovascular coupling within the PFC was found to underlie long-term enhancement of a mathematical cognitive task (Snowball et al., 2013). Chauvette et al. (2012) recently suggested that both endogenous as well as applied slow oscillatory activity may induce post-synaptic calcium-dependent plasticity. Taken together, the mechanisms through which presumed long-term storage occurred in the present study are still in need of elucidation.

Interestingly, contrary to our initial hypothesis, reference memory errors were not significantly reduced by SO-tDCS, indicating no detectable effect on long-term spatial memory. One possible reason for this could be that healthy, unimpaired animals were used, and a subtle intervention like SO-tDCS was not able to induce a further enhancement of intact consolidation of spatial memories in these animals. Possibly the application of a more challenging task would have been more sensitive. Along these lines, although the suppression of hippocampal ripples was shown to impair radial arm maze performance (Girardeau et al., 2009), REM sleep appears to be involved also (Legault et al., 2006). Thus, the essential involvement of slow oscillations for reference memory in the radial arm maze in the rodent has not been explicitly proven. It could also be speculated that effects of SO-tDCS, which are hypothesized to enhance the consolidation of context-independent features of the task, may be only detectable in a remote memory test conducted after a substantial delay extending beyond the 12 day training period. Bontempi et al. (1999) and Maviel et al. (2004) showed recruitment of neocortical areas and reduction of hippocampal activity in the radial maze task in such a remote memory test.

Another possible reason for the failure to modify reference memory could be the influence of food restriction on sleep quality: it is known that food deprivation can induce a decrease in TST, an increase in awakenings and reduced length of SWS episodes in rats (Jacobs and McGinty, 1971; Dewasmes et al., 1989). Similar findings were also reported in anorectic patients (Nobili et al., 2004). Since indications for the state-dependency of the effects of slow oscillatory electric stimulation on the electrophysiological level exist (Kirov et al., 2009; Fröhlich and McCormick, 2010; Ozen et al., 2010), one could speculate that impaired sleep quality or sleep fragmentation may have prevented SO-tDCS to further enhance spatial memory consolidation. Further studies employing different tasks are necessary to explore the impact of SO-tDCS on spatial memory consolidation in the rat model.

Taken together, the present study revealed that multiple daily sessions of SO-tDCS during NREM sleep impacted cortical network activity acutely and presumably also long-term responsiveness as well as behavior. Similar to a prior study in humans, post-stimulatory upper delta activity and the sleep-associated consolidation of task-inherent rules were enhanced. The decrease in EEG theta power commencing only after experimental day 8 is indicative for long-term effects of SO-tDCS on the cortical network, although the responsible mechanisms need as yet to be investigated. A missing enhancement of spatial memory consolidation as measured by reference memory errors may be related to task-specific features, making further studies necessary.

## Author contributions

Sonja Binder, Jan Born, and Lisa Marshall designed the study, Sonja Binder and Julia Rawohl conducted the experiment, Sonja Binder and Julia Rawohl analyzed the data, Sonja Binder, Jan Born, and Lisa Marshall wrote the paper.

### Conflict of interest statement

The authors declare that the research was conducted in the absence of any commercial or financial relationships that could be construed as a potential conflict of interest.
